# Primary immunodeficiency in infection-prone children in southern Sweden: occurrence, clinical characteristics and immunological findings

**DOI:** 10.1186/s12865-014-0031-6

**Published:** 2014-08-14

**Authors:** Nicholas Brodszki, Göran Jönsson, Lillemor Skattum, Lennart Truedsson

**Affiliations:** Children’s Hospital, Skåne University Hospital, Lund, SE-22185 Sweden; Department of Infectious Diseases, Skåne University Hospital, Lund University, Lund, SE-221 85 Sweden; Department of Laboratory Medicine, Section of Microbiology, Immunology and Glycobiology, Lund University, Lund, SE-221 85 Sweden

**Keywords:** Children, Complement, Immunodeficiency, Immunoglobulin, Immunoglobulin subclass, Lymphocyte

## Abstract

**Background:**

Primary immunodeficiency diseases (PIDs) comprise a heterogeneous group of disorders mainly characterized by increased susceptibility to infections. The aims of this study were to estimate the occurrence rate of PID in the paediatric (age ≤ 18 years) population of southern Sweden (approx. 265,000 children) and to describe their demographic, clinical and immunological characteristics. During a period of 4 years, in four paediatric speciality clinics in Skåne County in southern Sweden, children being seen for infections and fulfilling specific criteria were evaluated according to a predefined examination schedule. The initial analysis consisted of complete blood counts with analysis of lymphocyte subpopulations (T, B, NK cells), measurement of immunoglobulins (IgG, IgA, IgM, IgE and IgG subclasses), and assessment of the complement system (classical, alternative and lectin pathways). In addition, results of these immunological analyses in other children from the same area and time period were evaluated.

**Results:**

In total, 259 children (53.6% males) met the criteria and were included. The most common infection was recurrent otitis media. Immunological analyses results for about two thirds of the patients were outside age-related reference intervals. Further examination in this latter group identified 15 children with PID (9 males); 7 (2.7%) had genetically defined PID, representing 4 different diagnoses, and another 8 (3.1%) had a clinically defined PID - common variable immunodeficiency. No additional PID patient was identified from the evaluation of laboratory results in children not included in the study. The median age at diagnosis was 3.5 years (range 1–12 years).

**Conclusions:**

The occurrence rate of PID was about 4 new cases per year in this population. Several different PID diagnoses were found, and the application of specified criteria to identify PID patients was useful. In children who are prone to infection, the use of a predefined set of immunological laboratory analyses at their first examination was beneficial for early identification of patients with PID.

## Background

Primary immunodeficiency diseases (PIDs) are a heterogeneous group of disorders mainly characterized by severe and recurrent infections but also increased susceptibility to autoimmune conditions and malignancies. PIDs are relatively rare in the general population, affecting between 1 in 500 to 1 in 500,000 individuals, with varying degrees of ascertainment in different countries [1-5]. Most PIDs are hereditary, and many of these diseases present during infancy or in early childhood [6]. Because recurrent or severe infections are important clinical characteristics of PID, early detection of PID is critical, before these infections compromise a patient’s general condition. A correct and early diagnosis is needed for optimal treatment and for timely family genetic counselling [7].

Children suffer more often than adults from infections, and infection frequency is higher in younger age groups. This pattern is especially true for children who are regularly exposed to infectious agents, such as those attending institutional day care [8]. Because few children have a demonstrable PID, it can be challenging for the investigating physician to identify in this group the relatively rare child with an immunodeficiency. Based on estimations of frequency of several forms of PID, a considerable proportion of these patients do not receive a correct diagnosis in a timely fashion [9,10]. From our own experience, we believe that there are more children with PID than published statistics would suggest [11].

The objectives of this study were to estimate the occurrence rate of PID in the paediatric population of southern Sweden and to describe their demographic, clinical and immunological characteristics. Children in a defined area in southern Sweden who needed health care for infections and fulfilling specific criteria were evaluated according to a predefined examination schedule including a set of laboratory analyses. In total, 15 children with PID, representing five diagnoses, were identified during a 4-year period in a paediatric population of around 265,000 (ages ≤18 years).

## Methods

### Patients

The study area consisted of Skåne, the southernmost county in Sweden with a population of 1.2 million. Region Skåne is a regional public body with administrative and financial responsibility for the health of the inhabitants and for providing medical and dental services. In Region Skåne, there are four major paediatric clinics where children with suspected PID are evaluated, and all diagnostic immunological analyses are performed at one accredited laboratory (Clinical Immunology and Transfusion Medicine, Laboratory Medicine Skåne, Lund, Sweden).

The children in the study were referred to the four clinics from either general practitioners or paediatricians, and the main reason for referral was a history of recurrent infections. During a period of 4 years, from October 2007 to November 2011, the children presenting for health care for frequent infections at these four clinics were evaluated according to a predefined examination schedule. The paediatric population in Skåne (≤18 years), in the middle of the study period (December 2009), was 268,439 (13.1% of all children in Sweden).

The criteria for evaluation and thus inclusion in the study were based primarily on the “10 Warning Signs” (Table 1) defined by the Swedish Physicians Working with Immunodeficiency (SLIPI) [12]. Because other clinical symptoms can raise the suspicion of PID, we extended the inclusion criteria beyond the 10 warning signs to incorporate the following additional symptoms: recurrent fever or fever with duration of more than 6 weeks, and an excessive number of upper respiratory tract infections (>8 per year) [8]. The examination schedule consisted of medical history, physical examination and a predefined set of laboratory analyses. Blood samples were stored to permit later analysis of disease genes, specific antibodies and other immune molecules.

**Table 1:** **Inclusion criteria for evaluation of primary immunodeficiency disease** *

1)	Six or more new ear infections within 1 year; otitis with complications such as chronic perforation or mastoiditis
2)	Two or more serious sinus infections within 1 year
3)	Infections that do not heal as expected during antibiotic treatment
4)	Two or more pneumonias within 1 year
5)	Failure of an infant to gain weight or grow normally
6)	Recurrent, deep skin infections or organ abscesses
7)	Chronic severe oral or cutaneous candidiasis
8)	Infections caused by unusual microbial agents and/or with unusual localization
9)	Two or more invasive infections such as osteomyelitis, meningitis or sepsis
10)	A family history of PID
11)	Recurrent fever or fever lasting more than 6 weeks
12)	Excessive number of upper respiratory tract infections (more than 8 per year)

*The first 10 criteria are identical to the “10 Warning Signs” given by Swedish Physicians Working with Immunodeficiency (SLIPI) [12].

Ethical permission for the study was obtained from the Regional Ethical Review Board, Lund, Sweden (LU 300/2007). Written informed consent for participation in the project and for storage of the blood samples for later analysis was obtained from all patients and their custodians. Permission for storage of DNA data was obtained from the local division of The Swedish Data Inspection Board.

To ensure that no children with symptoms suggestive of PID were missed, the results of the same immunological laboratory analysis as included in the predefined set, performed in other children (ages 0–18 years) from the same area, during the same time period, were also analysed. This additional evaluation was possible because all of these analyses were performed at the same Lund laboratory.

### Laboratory analysis

Complete blood counts were measured using standard laboratory methods. Analysis of lymphocyte subpopulations defined by expression of CD3 (T cells), CD4 (T helper cells), CD8 (T cytotoxic cells), CD19 (B cells) and CD16/56 (NK cells) was performed by flow cytometry. Serum immunoglobulin (Ig) concentrations including IgG, IgA, IgM, IgE and IgG subclasses were measured using nephelometry. The functions of classical, alternative and lectin pathways of complement in serum were assessed with the Wieslab® Complement system Screen kit (Euro Diagnostica, Malmö, Sweden) [13] and concentrations of the components C3 and C4 by nephelometry, while C1q and properdin levels were determined with electroimmunoassay and mannose-binding lectin (MBL) level was determined with ELISA. Further analysis of complement components was performed in patients with abnormal results in the functional complement assays.

Because all immunological analyses in southern Sweden are performed at our laboratory, we have access to the results for all children tested during the study period in this area. The immunological tests are offered by default as certain combinations (clusters) at our laboratory and are recommended for investigating patients with infection proneness, thus allowing the possibility of comparing the results of any patient with the study patients. The tests performed for patients examined outside the study were either only one or a combination of the following clusters: Ig, Ig subclasses, complement and lymphocyte subpopulations. Results from these immunological analyses in children from departments where they were investigated for reasons other than suspicion of PID (*e.g.* oncology, transplantation, rheumatology and nephrology) were excluded.

## Results

### Demographic characteristics

**Figure 1:**
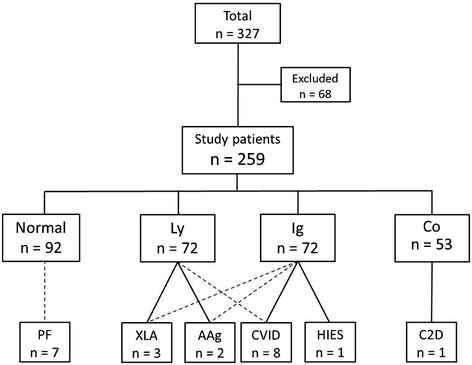
**Flow chart and summarized results for patients included in the study.** Of 327 patients enrolled, 68 were excluded because they did not meet the inclusion criteria or they had a known disease predisposing for infections. For the remaining 259 patients, the number of patients with immunological laboratory results within (Normal) or outside the reference intervals for immunoglobulins (Ig), lymphocytes (Ly) and complement (Co) are given. The number of patients and type of PID found in those with deviating results are also shown. The analyses performed are stated in the Methods section; AAg, autosomal agammaglobulinaemia; C2D, complement C2 deficiency; CVID, common variable immunodeficiency; HIES, hyper-IgE syndrome; PF, periodic fever, aphthous stomatitis, pharyngitis and adenitis; XLA, X-linked agammaglobulinaemia.

During a 4-year period, from October 2007 to November 2011, a total of 327 patients were evaluated in accordance with our predefined schedule (Figure 1). Of these patients, 41 did not meet any of the inclusion criteria. Nevertheless, the clinicians responsible for these patients considered that an immunologic assessment should be done and used our schedule to start the evaluation. Another 27 patients were also excluded from the final analysis because of an already known underlying chronic or genetic disease (*e.g*. 22q11 deletion syndrome or cystic fibrosis) that predisposes to infections.

**Figure 2:**
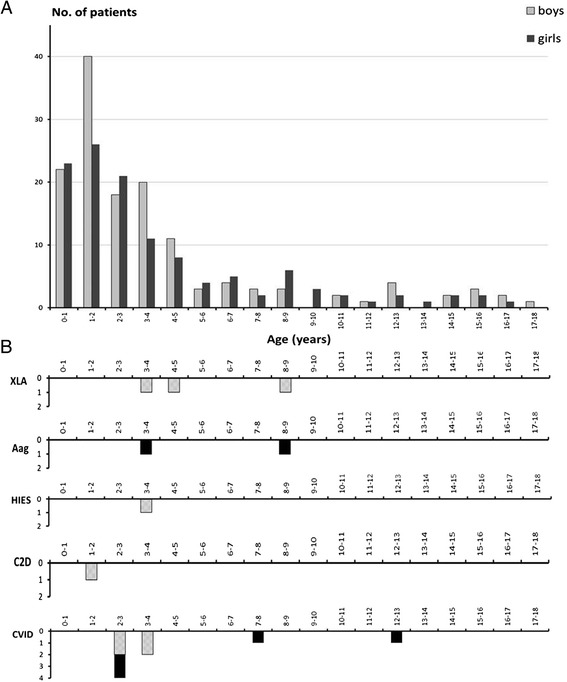
**Age distribution of the patients. A**. Age distribution of the included 259 study patients (120 female and 139 males). **B**. Age distribution of patients with a PID diagnosis. Abbreviations of diagnoses as in Figure 1.

The age interval of the 259 children included was from 1 month to 17 years with a median age of 28 months. There were 139 (53.6%) males and 120 females (46.4%) (Figure 2). The majority (n = 240) were Caucasians of European descent; 17 patients were of Middle-Eastern origin and two were of Asian origin.

According to the medical records, the study patients were characterized by recurrent, but not always severe, infections. In total, 52 patients fulfilled more than one criterion, 43 had two criteria, 6 had three criteria and 3 had four criteria fulfilled. The most common reason for investigation was recurrent otitis media (n = 102 patients). As expected, a clear relationship with age was seen for the ear infections, with the majority of these patients just over age 2 years (median 25 months, range 6 months to 9 years). The second most common infection type was pneumonia (n = 52) followed by abnormal number of upper respiratory tract infections (n = 35).

During the same 4-year period, another 2442 unique patients with infection proneness were analysed with one or two of the laboratory clusters described in the Methods. Only 19 patients were analysed with the same three clusters as the study patients, and all of these had normal results.

### Immunological laboratory findings

Only about one third of the 259 patients (35.5%) had results of the immunological analyses within the age-matched reference intervals. Many of the study patients with abnormal laboratory results had several abnormal findings (Figure 1). Seven patients (2.7%) had a genetically identified and another eight patients a clinically defined PID. As seen in Figure 1, all patients who received a PID diagnosis had abnormal laboratory results. The results of the immunological analyses in the study patients and in other children with infections from county of Skåne are presented in Table 2. None of the patients outside the study received a PID diagnosis.

**Table 2:** **Children with suspected primary immunodeficiency disease with results of immunological analysis outside reference intervals**

		**Study (n = 259)** ^**a**^ **No. (%)**	**Excluded (n = 41)** ^**b**^ **No. (%)**	**Skåne** ^**c**^ **county No. (%)**	
Immunoglobulins	IgG	21 (8.1)	0	16 (1.5)	
	IgA	0	0	1 (0.1)	n = 1056
	IgM	21 (8.1)	0	37 (3.5)	
Immunoglobulin subclasses	IgG_1_	0	0	10 (1.0)	n = 1080
	IgG_2_	24 (9.2)	0	59 (5.4)	
	IgG_3_	4 (1.4)	0	0	
	IgG_1_ + IgG_2_	1 (0.4)	0	3 (0.3)	
	IgG_1_ + IgG_3_	0	0	3 (0.3)	
	IgG_2_ + IgG_3_	1 (0.4)	0	1 (0.1)	
Lymphocytes	T cells	34 (13.1)	5 (12.2)	17 (4.8)	n = 348
	T helper cells	2 (0.7)	0	1 (0.3)	
	T cytotoxic cells	4 (1.4)	2 (4.8)	0	
	B cells	19 (7.3)	1 (2.4)	4 (1.1)	
	NK cells	13 (5.0)	0	13 (3.7)	
Complement	Classical pathway	9 (3.5)	1 (2.4)	26 (1.7)	n = 1473
	Alternative pathway	5 (1.9)	0	14 (0.9)	
	Lectin pathway	63 (24.3)	4 (9.6)	26 (18.4)	n = 141

^a^Patients fulfilling the study inclusion criteria.

^b^The 41 patients not fulfilling the study inclusion criteria but with results from the predefined set of immunological analyses (the 27 patients excluded because of known genetic diseases are omitted).

^c^Other patients with infections in which immunological analyses were performed.

#### Immunoglobulin

As expected, considering the median age of the children, the most common finding regarding results of Ig analysis was IgG subclass deficiency, defined as significantly decreased concentration of one or more subclasses of IgG in a patient with normal IgG concentration [14]. The distribution of the different deficiencies among the 32 patients and their treatment are given in Table 3. No patients had selective IgA deficiency, defined as a serum IgA level <0.07 g/L and age over 4 years.

**Table 3:** **Patients with IgG subclass deficiency**

**Deficiency** ^**a**^	**No. of patients**	**Treatment**
**IgG1**	0	-
**IgG2**	23	Ig infusion: 5 patients
**IgG3**	4	-
**IgG4** ^**b**^	0	-
**IgG1 + IgG2**	1	Ig infusion
**IgG1 + IgG3**	0	-
**IgG2+ IgG3**	1	Ig infusion
**IgG2 + IgA**	3	Ig infusion: 1 patient
**IgG3 + low IgA, IgM**	1	Ig infusion

^a^Defined as a significant decrease in serum concentration of one or more subclasses of IgG when total IgG concentration is normal (6). Values below age-matched reference intervals were considered as significantly decreased.

^b^Age >12 years.

#### Lymphocytes

The measurement of complete blood counts did not lead to identification of any additional PID cases that were not also found by the immunological analyses. More than one in four of the patients (27.4%) had an abnormal result in the lymphocyte analyses (Figure 1). Many of these patients had, for their age, a higher than expected number of viral upper respiratory tract infections as their only clinical characteristic. Analysis of the lymphocyte subpopulations revealed no patients with total deficiency of T cells, but relative to the age-matched reference interval, there were 34 patients with low levels.

**Figure 3:**
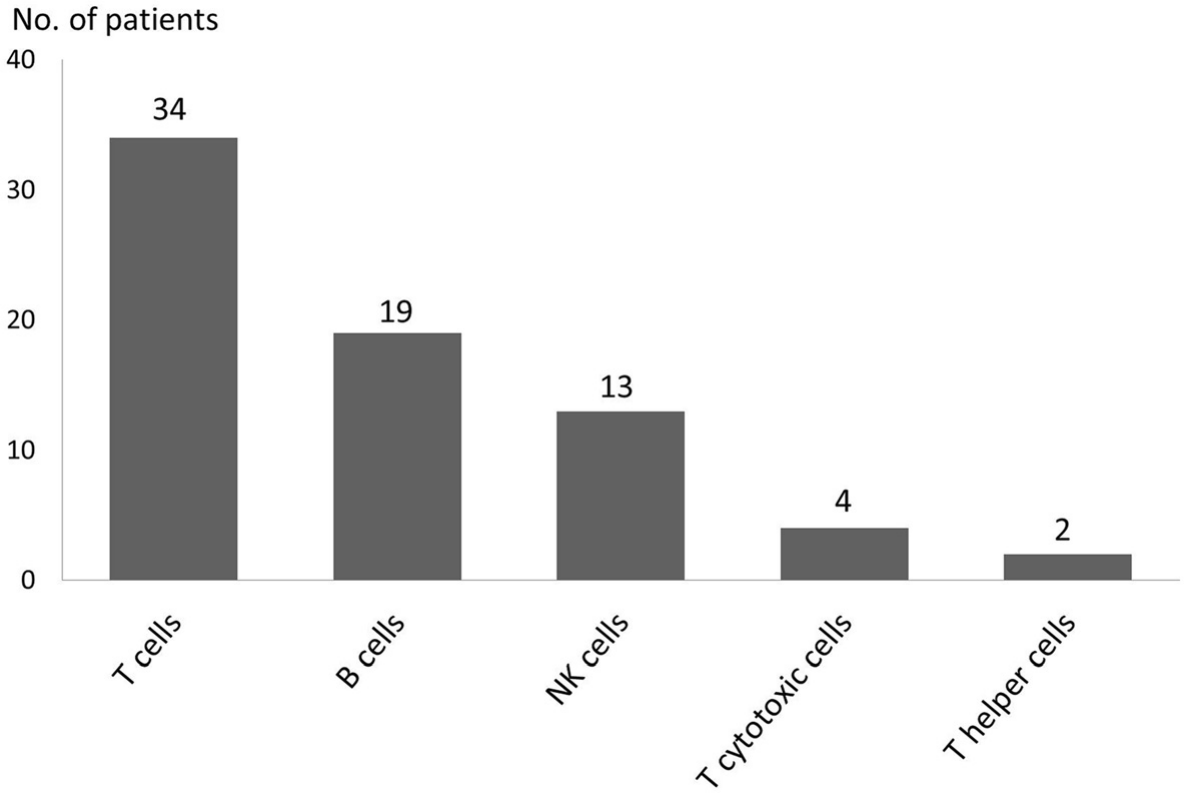
**Results of the lymphocyte analysis.** Number of patients with an isolated subnormal number of T, B and NK cells shown together with the number of patients with an isolated low number of T cell subpopulations. In total, 72 of 259 patients had lymphocyte analysis results below reference intervals.

In total, 24 patients (63% males) had low numbers of circulating B cells, some of them in combination with variably low numbers of T cells. All of these patients had normal levels of the Ig isotypes measured. The numbers of patients with isolated aberrancies in any of the lymphocyte subpopulations are shown in Figure 3.

#### Complement

About one fifth of the patients (20.5%) did not have all results of the complement analyses within the reference intervals (Figure 1). Nine patients had decreased function of the classical pathway without a common clinical denominator. Five patients showed decreased function of the alternative pathway. Two of these could be explained by low levels of properdin, but three of them were unexplained, and there was no common clinical association. Nineteen patients had low levels of C1q without any detectable C1q antibodies. Of these, seven are currently on Ig infusions. Although they received Ig for reasons other than the low C1q (two because of X-linked agammaglobulinaemia [XLA], four because of common variable immunodeficiency [CVID] and one for IgG2 deficiency), this finding confirms the described direct association of low C1q levels with low IgG levels [15], that C1q concentration is age-dependent and that small children have lower levels [16]. Low levels of properdin were found in 15 patients.

MBL deficiency, defined as MBL concentration <100 μg/L, was a common finding, identified in 63 cases (24.3%). The high incidence of MBL deficiency is not explained by the age of patients [17] or ancestry because the majority of the patients were of Scandinavian ancestry, and the supposed frequency in this group is approximately 10% [18]. The main reason for investigation of these children was otitis media, thus confirming earlier observations that the lectin pathway plays a role in upper airway infections during early childhood [19]. The importance of MBL deficiency alone is questionable, and we do not here refer to the patients with this laboratory test result as having an immunodeficiency.

### Clinical outcome

The patients with laboratory results or clinical symptoms that indicated a PID disease have been followed for a period of between 2 to 6 years. We have identified PID in 15 children (Figure 2B), seven with genetically defined PID disease and eight with a clinically defined PID diagnosis [1].

#### Genetically defined PID

Three patients were diagnosed with XLA with defects in the *Bruton tyrosine kinase* gene. One patient with IgG2 deficiency and a defect in the complement classical pathway was diagnosed with complete C2 deficiency type 1. One patient with high IgE serum concentration was confirmed to have a mutation in the *STAT3* gene, leading to the diagnosis of Hyper-IgE syndrome. Finally, two female patients with no B cells had mutations in the *IGLL1* gene, and their diagnosis was autosomal recessive agammaglobulinaemia. During the study period, no patients with severe combined immunodeficiency (SCID) were identified in the county of Skåne.

#### Clinically defined PID

In this cohort, several patients had well-characterized PID but without a known genetic defect. Thus, we diagnosed eight patients with CVID, defined as a patient older than age 2 years with IgG and IgA and/or IgM levels two standard deviations below the mean for age and an absence of other detectable immunodeficiency [20,21]. Nine of the patients could be classified as having transient hypogammaglobulinaemia of infancy or unclassified hypogammaglobulinaemia as recently defined by the European Society for Immunodeficiency (ESID) [22]. However, we do not here specify these as patients with PID because the study design did not allow us to distinguish between these two groups and the significance of this deficiency is questionable.

#### Other diagnosis than PID

During the follow-up, seven of the tested patients fulfilled the criteria for one of the known autoinflammatory syndromes [23], for a diagnosis of periodic fever, aphthous stomatitis, pharyngitis and adenitis (known as PFAPA). None of these patients showed any immunological aberrations, emphasizing the importance of evaluating patient symptoms.

## Discussion

Early diagnosis and adequate therapy are the keys to survival and a better quality of life for patients with PID. To identify all children with PID in the study area, we focused on the infection proneness typical for PID and used specified inclusion criteria. Similar warning signs have been implemented by ESID after initiation of the present investigation [22]. Comparable warning signs are used worldwide and have been suggested by the Jeffrey Modell Foundation [24], the United States Immunodeficiency Network and the Immune Deficiency Foundation [25]. However, the warning signs carry limitations as a screening tool because they may fail to identify some patients with serious PIDs, as recently reported [26,27]. The warning sign of having a family member with a known immunodeficiency is advocated as one of the three most important signs for identifying patients with PID [27]. In our study, however, only four children met this criterion, most likely because the ethnic background of our population suggests a very low percentage of consanguinity. However, one patient with a genetic deficiency was discovered through this warning sign, indicating its importance. In our study, recurrent otitis media was the most common type of infection prompting the study patients to seek health care, which is explained by the age distribution characteristic for this diagnosis [28].

The children included were all investigated with a predefined standardized “starting kit” of immunological analyses that are generally available and easy to perform. We believe this approach would help physicians with the initial evaluation of patients suspected of having PID and thus lead to an early diagnosis; indeed, all PID patients we found had laboratory results outside reference intervals. However, because these analyses also were performed in other children not included in the study, we used available laboratory data to ensure that no PID patients were missed. A certain number of results in these other children were not within the normal reference ranges, but because no additional patient with PID was found, this outcome clearly suggests that all PID children in the area were identified. Also, none of the test results led to the referral of any patient to any of the four paediatric clinics.

The information regarding PID occurrence in Sweden is scarce. In a previous study based on a survey of symptomatic PID in children in Sweden during a 6-year period in the 1970s, 174 cases were found [29]. Considering the population in Sweden of about 9.5 million, our number of 15 cases during 4 years in a population of 1.2 million is of the same magnitude. In our cohort, a relatively low predominance of boys was seen, with a male/female ratio of 1.1/1, compared to previously described cohorts in which this ratio was 1.4/1 to 2/1 [29,30]. A possible explanation for our results is the different recruitment method because we included all patients with symptoms suggestive of PID who sought health care.

Regarding the 32 patients with IgG subclass deficiencies, our findings are similar to earlier observations [31]. The significance of IgG subclass deficiency in childhood is controversial, and we thus did not include these patients in the number of PID patients. However, as seen in Table 3, these patients still need treatment, and checking for this deficiency should be done. The absence of selective IgA deficiency was noted but is reasonably explained by the low number of patients older than 4 years. We chose not to include the results from analysis of specific antibodies in this report because the Swedish official immunization schedule for children was changed during the study, making interpretation complex, but the aim is to include these findings in a forthcoming publication.

Considering the results of the lymphocyte analyses, a fair proportion of the patients showed a subnormal concentration of one or several lymphocyte subpopulations. Clearly, the number of such patients found in our study is higher than expected, but the results could not be associated with any particular diagnosis or symptoms. Changes in distribution of lymphocyte subpopulations occur often in infections. The deviations noted could be temporary, but the design of the study did not make this possible to verify. The justification of the lymphocyte analysis, however, is the need to detect severe PIDs like SCID and XLA without delay. Further studies are warranted to define the role for these analyses of the immune system in this patient group.

A few children showed reduced activity in the functional test for the alternative complement pathway without any known cause. This outcome is in line with previous observations that defective function in this pathway occasionally is seen in sera from newborns [32]. An interesting finding was the great number of patients with a non-functioning lectin pathway. Eleven (17%) of the patients with MBL deficiency were given treatment (antibiotic prophylaxis). Of these, 4 patients had no other immunological analysis with discrepant result, suggesting that MBL deficiency may be the sole reason for their recurrent infections. Considering the percentage of patients in need of treatment, these findings are highly relevant for the practicing clinician. There is also convincing evidence in the literature that MBL deficiency is associated with increased morbidity risk when immunity is co-compromised by another defect [33], as in cystic fibrosis [34], although the nature of the association may vary [18]. Another example is that the combination of C2 deficiency and MBL deficiency appears to increase infection proneness [35]. Thus, MBL deficiency may be regarded as a disease modifier, but some patients with this deficiency and no other known defect in their immune system still require treatment.

We argue therefore that testing the function of the complement system should be done early during an immunological evaluation. The defects in the complement system are in general not possible to correct, but many complications are avoidable if the defect is identified early enough. In C2 deficiency, vaccination against the encapsulated bacteria *S. pneumoniae* and *H. influenzae* can give rise to antibodies and opsonisation, clearly suggesting increased protection [36]. Vaccination against other encapsulated bacteria such as meningococci is also recommended in complement deficiency states.

We identified eight patients with CVID, which is fewer than expected from previously published studies [37]. CVID is usually diagnosed in the second or third decade of life, but conform to a recent study 33.7% of CVID patients present before the age of 10 years [38], and there is a described earlier peak of diagnosis at approximately age 8 years [39]. The diagnosis of CVID before age 6 years is questionable because of a possible delay in immunologic maturation and because transient hypogammaglobulinaemia of infancy may persist in some children [40]. We think that our low numbers are the result of the fact that the mean age in this cohort of patients was low and the follow-up time for many of them was not long enough.

The fact that no patients with SCID were identified in southern Sweden during this period is in concordance with the published statistics considering the frequency of SCID [6] because the number of children born every year in Skåne is approximately 12,500.

A limitation of our study is that because there are PIDs with a non-infectious phenotype, they might have been missed given that the warning signs do not contain that type of symptom [26,27]. Another weakness is that from our data, it is impossible to estimate the prevalence of PID in the county of Skåne. The study period was only 4 years; the age of onset of many PIDs is very broad, however, and some patients might not yet have presented with symptoms. Many of the patients included underwent laboratory analysis only once, so that an abnormal value might be normal if the analysis were repeated; the contrary might be true for a normal value, as well. Some abnormal values are likely explained by an ongoing infection and not an underlying PID. However, this is the situation when the sick child is seen by the physician, and our results clearly show the usefulness of the standardized starting set of immunological laboratory tests in combination with the clinical warning signs. Saving blood samples, including cells, for DNA analysis makes it possible to add genetic analysis without a repeated sampling. Also, there may be an opportunity of diagnosing an immunodeficiency disease that has yet to be identified but might be characterized in the near future.

In 2012, after the start of our study, an updated version of a diagnostic protocol for screening for PID was published by the ESID Clinical Working Party [41]. We are aware that the multi-stage protocol differs to some extent from ours. However, we consider that our results are relevant because our protocol allows patient identification early in the diagnostic process.

## Conclusions

The use of specified criteria in children presenting for health care for infections and evaluation according to a predefined examination schedule, including a set of laboratory analyses, is useful for identifying children with PID. In the studied paediatric population of around 265,000 (ages ≤18 years) in southern Sweden, about four new cases of PID per year were seen provided that unclassified hypogammaglobulinaemia and MBL deficiency were not included as PID diagnoses.

## Abbreviations

CVID: Common variable immunodeficiency
Ig: Immunoglobulin
MBL: Mannose-binding lectin
PFAPA: Periodic fever, aphthous stomatitis, pharyngitis and adenitis
PID: Primary immunodeficiency disease
SCID: Severe combined immunodeficiency
XLA: X-linked agammaglobulinaemia

## References

[1] Chapel H (2012). Classification of primary immunodeficiency diseases by the International Union of Immunological Societies (IUIS) Expert Committee on Primary Immunodeficiency 2011. Clin Exp Immunol.

[2] Al-Herz W, Bousfiha A, Casanova JL, Chapel H, Conley ME, Cunningham-Rundles C, Etzioni A, Fischer A, Franco JL, Geha RS, Hammarstrom L, Nonoyama S, Notarangelo LD, Ochs HD, Puck JM, Roifman CM, Seger R, Tang ML (2011). Primary immunodeficiency diseases: an update on the classification from the international union of immunological societies expert committee for primary immunodeficiency. Front Immunol.

[3] Gathmann B, Grimbacher B, Beaute J, Dudoit Y, Mahlaoui N, Fischer A, Knerr V, Kindle G, Party ERW (2009). The European internet-based patient and research database for primary immunodeficiencies: results 2006–2008. Clin Exp Immunol.

[4] Fischer A (2007). Human primary immunodeficiency diseases. Immunity.

[5] Boyle JM, Buckley RH (2007). Population prevalence of diagnosed primary immunodeficiency diseases in the United States. J Clin Immunol.

[6] Notarangelo LD, Fischer A, Geha RS, Casanova JL, Chapel H, Conley ME, Cunningham-Rundles C, Etzioni A, Hammartrom L, Nonoyama S, Ochs HD, Puck J, Roifman C, Seger R, Wedgwood J (2009). Primary immunodeficiencies: 2009 update. J Allergy Clin Immunol.

[7] Folds JD, Schmitz JL (2003). 24. Clinical and laboratory assessment of immunity. J Allergy Clin Immunol.

[8] Gruber C, Keil T, Kulig M, Roll S, Wahn U, Wahn V (2008). History of respiratory infections in the first 12 yr among children from a birth cohort. Pediatr Allergy Immunol.

[9] Shearer WT, Cunningham-Rundles C, Ochs HD (2004). Primary immunodeficiency: looking backwards, looking forwards. J Allergy Clin Immunol.

[10] Buckley RH (2006). Primary immunodeficiency or not? Making the correct diagnosis. J Allergy Clin Immunol.

[11] Sjoholm AG, Jonsson G, Braconier JH, Sturfelt G, Truedsson L (2006). Complement deficiency and disease: an update. Mol Immunol.

[12] **10 Warning signs.** ᅟ [http://slipi.nu/klinik/Medicinsk_info.shtml]

[13] Seelen MA, Roos A, Wieslander J, Mollnes TE, Sjoholm AG, Wurzner R, Loos M, Tedesco F, Sim RB, Garred P, Alexopoulos E, Turner MW, Daha MR (2005). Functional analysis of the classical, alternative, and MBL pathways of the complement system: standardization and validation of a simple ELISA. J Immunol Methods.

[14] Herrod HG (1993). Clinical significance of IgG subclasses. Curr Opin Pediatr.

[15] Plebani A, Notarangelo LD, Duse M, Avanzini A, Massa M, Ugazio AG (1984). Serum IgG levels and complement activity in hypogammaglobulinaemic patients under substitution therapy. Clin Exp Immunol.

[16] Prellner K, Sjoholm AG, Truedsson L (1987). Concentrations of C1q, factor B, factor D and properdin in healthy children, and the age-related presence of circulating C1r-C1s complexes. Acta Paediatr Scand.

[17] Sallenbach S, Thiel S, Aebi C, Otth M, Bigler S, Jensenius JC, Schlapbach LJ, Ammann RA (2011). Serum concentrations of lectin-pathway components in healthy neonates, children and adults: mannan-binding lectin (MBL), M-, L-, and H-ficolin, and MBL-associated serine protease-2 (MASP-2). Pediatr Allergy Immunol.

[18] Carlsson M, Sjoholm AG, Eriksson L, Thiel S, Jensenius JC, Segelmark M, Truedsson L (2005). Deficiency of the mannan-binding lectin pathway of complement and poor outcome in cystic fibrosis: bacterial colonization may be decisive for a relationship. Clin Exp Immunol.

[19] Schlapbach LJ, Latzin P, Regamey N, Kuehni CE, Zwahlen M, Casaulta C, Aebi C, Frey U (2009). Mannose-binding lectin cord blood levels and respiratory symptoms during infancy: a prospective birth cohort study. Pediatr Allergy Immunol.

[20] Bonilla FA, Bernstein IL, Khan DA, Ballas ZK, Chinen J, Frank MM, Kobrynski LJ, Levinson AI, Mazer B, Nelson RP, Orange JS, Routes JM, Shearer WT, Sorensen RU (2005). Practice parameter for the diagnosis and management of primary immunodeficiency. Ann Allergy Asthma Immunol.

[21] Conley ME (1999). Diagnostic guidelines–An International Consensus document. Clin Immunol.

[22] **10 Warning Signs of PID - General.** ᅟ [http://esid.org/Working-Parties/Clinical/Resources]

[23] Tunca M, Ozdogan H (2005). Molecular and genetic characteristics of hereditary autoinflammatory diseases. Curr Drug Targets Inflamm Allergy.

[24] **10 Warning Signs.** ᅟ [http://www.info4pi.org/library/educational-materials/10-warning-signs]

[25] **Resource Links.** ᅟ [http://usidnet.org/resource-links/]

[26] Arkwright PD, Gennery AR (2011). Ten warning signs of primary immunodeficiency: a new paradigm is needed for the 21st century. Ann N Y Acad Sci.

[27] Subbarayan A, Colarusso G, Hughes SM, Gennery AR, Slatter M, Cant AJ, Arkwright PD (2011). Clinical features that identify children with primary immunodeficiency diseases. Pediatrics.

[28] Teele DW, Klein JO, Rosner B (1989). Epidemiology of otitis media during the first seven years of life in children in greater Boston: a prospective, cohort study. J Infect Dis.

[29] Fasth A (1982). Primary immunodeficiency disorders in Sweden: cases among children, 1974–1979. J Clin Immunol.

[30] **Statistics.** ᅟ [http://esid.org/Working-Parties/Registry]

[31] Meulenbroek AJZW (2000). Human IgG subclasses: Useful diagnostic markers for immunocompetence.

[32] Truedsson L: *Studies on complement and anti-immunoglobulins. Development of hemolysis in gel and immunoenzyme techniques.* Lund University, Department of Medical Microbiology; 1984. PhD thesis.

[33] Aittoniemi J, Baer M, Soppi E, Vesikari T, Miettinen A (1998). Mannan binding lectin deficiency and concomitant immunodefects. Arch Dis Child.

[34] Garred P, Pressler T, Madsen HO, Frederiksen B, Svejgaard A, Hoiby N, Schwartz M, Koch C (1999). Association of mannose-binding lectin gene heterogeneity with severity of lung disease and survival in cystic fibrosis. J Clin Invest.

[35] Jonsson G, Oxelius VA, Truedsson L, Braconier JH, Sturfelt G, Sjoholm AG (2006). Homozygosity for the IgG2 subclass allotype G2M(n) protects against severe infection in hereditary C2 deficiency. J Immunol.

[36] Jonsson G, Lood C, Gullstrand B, Holmstrom E, Selander B, Braconier JH, Sturfelt G, Bengtsson AA, Truedsson L (2012). Vaccination against encapsulated bacteria in hereditary C2 deficiency results in antibody response and opsonization due to antibody-dependent complement activation. Clin Immunol.

[37] Martin-Nalda A, Soler-Palacin P, Espanol Boren T, Caragol Urgelles I, Diazde Heredia Rubio C, Figueras Nadal C (2011). [Spectrum of primary immunodeficiencies in a tertiary hospital over a period of 10 years]. An Pediatr (Barc).

[38] Gathmann B, Mahlaoui N, for C, Gerard L, Oksenhendler E, Warnatz K, Schulze I, Kindle G, Kuijpers TW, Dutch WID, van Beem RT, Guzman D, Workman S, Soler-Palacin P, De Gracia J, Witte T, Schmidt RE, Litzman J, Hlavackova E, Thon V, Borte M, Borte S, Kumararatne D, Feighery C, Longhurst H, Helbert M, Szaflarska A, Sediva A, Belohradsky BH, Jones A (2014). Clinical picture and treatment of 2212 patients with common variable immunodeficiency. J Allergy Clin Immunol.

[39] Yong PL, Orange JS, Sullivan KE (2010). Pediatric common variable immunodeficiency: immunologic and phenotypic associations with switched memory B cells. Pediatr Allergy Immunol.

[40] Ogershok PR, Hogan MB, Welch JE, Corder WT, Wilson NW (2006). Spectrum of illness in pediatric common variable immunodeficiency. Ann Allergy Asthma Immunol.

[41] de Vries E (2012). Patient-centred screening for primary immunodeficiency, a multi-stage diagnostic protocol designed for non-immunologists: 2011 update. Clin Exp Immunol.

